# Domain Evolution of Vertebrate Blood Coagulation Cascade Proteins

**DOI:** 10.1007/s00239-022-10071-3

**Published:** 2022-10-01

**Authors:** Abdulbaki Coban, Erich Bornberg-Bauer, Carsten Kemena

**Affiliations:** 1 Institute for Evolutionary Biology, WWU Münster, Münster, Germany; 2 Max Planck-Institute for Biology Tuebingen, Tübingen, Germany

**Keywords:** Blood coagulation, Protein domains, Modular evolution, Hemolymph clotting

## Abstract

Vertebrate blood coagulation is controlled by a cascade containing more than 20 proteins. The cascade proteins are found in the blood in their zymogen forms and when the cascade is triggered by tissue damage, zymogens are activated and in turn activate their downstream proteins by serine protease activity. In this study, we examined proteomes of 21 chordates, of which 18 are vertebrates, to reveal the modular evolution of the blood coagulation cascade. Additionally, two Arthropoda species were used to compare domain arrangements of the proteins belonging to the hemolymph clotting and the blood coagulation cascades. Within the vertebrate coagulation protein set, almost half of the studied proteins are shared with jawless vertebrates. Domain similarity analyses revealed that there are multiple possible evolutionary trajectories for each coagulation protein. During the evolution of higher vertebrate clades, gene and genome duplications led to the formation of other coagulation cascade proteins.

**Supplementary Information:**

The online version contains supplementary material available at 10.1007/s00239-022-10071-3.

## Introduction

Coagulation (blood clotting) is the process of gel formation of the blood at injured and/or damaged tissue sites. Under normal conditions, blood circulates through arteries, capillaries and veins but responds to tissue damage by clot formation. Therefore, it is essential to have a rapid and effective coagulation mechanism to prevent blood loss as well as undesirable coagulation within veins (thrombosis). Clotting is regulated by a complex cascade which involves more than two dozen proteins including serine proteases (Doolittle (2009)).

The cascade is divided into three pathways: the intrinsic pathway, the extrinsic pathway and the common pathway (Grover and Mackman (2019)). The intrinsic pathway is triggered by the internal trauma in the blood vessels. Factor XII (FXII) is activated by exposure to endothelial collagen which then can activate downstream proteins (Chaudhry et al (2021)). As for the extrinsic pathway, damaged endothelium of extravascular cells releases tissue factor (TF) into the blood stream and TF acts as a cofactor of activated Factor VII (FVII) to enhance its protease activity (Smith et al (2015)). These two pathways merge into a common pathway involving the Factor X (FX) and Factor V (FV) complex. This common pathway results in fibrin clots on the wound site (Supplementary Figure S1).

More than half of the proteins which belong to the blood coagulation cascade are serine proteases which are highly similar to each other in terms of function and structure: They serve as activators/deactivators of downstream proteins with their protease activity and possess the same or similar domain arrangements (Doolittle (2009)). It is clear that the serine proteases share common ancestor(s) due to the domain similarities not only in their terminal protease domains but also in their auxiliary Gla, EGF, and Kringle domains (Doolittle (2009); Ponczek et al (2012)).

Serine proteases FIX, FX, Protein C and Protein Z have identical domain arrangements: *Gla – EGF – Fxa inhibition – Trypsin*. Similarly, cofactors of the system, FV and FVIII, also share similar domain arrangements*: Cu-oxidase (4–6 repeats) – F5 F8 type C*. Finally, Plasma Kallikrein and Factor XI (FXI) also share the same domain organization: *PAN 1 (*× *4) – Trypsin*. Gene duplications, fusions, fissions and terminal losses can lead to novel domain arrangements (Bornberg-Bauer and Albà (2013); Cromar et al (2014); Klasberg et al (2018)). Therefore, investigating the changes in domain arrangements can reveal the modular evolution of the blood coagulation cascade (Patthy (1985)).

Analogous to blood coagulation in vertebrates, Arthropods have a hemolymph clotting system to prevent hemolymph loss and provide immunity (Theopold et al (2002, 2004)). Even though both systems possess proteins such as serine proteases, cofactors, and clot stabilizers which are similar in terms of their functions, clotting mechanisms differ between the blood coagulation and the hemolymph clotting systems (Theopold et al (2002, 2004)). While the sequence similarities between the components of these two systems are low, it is argued that both systems share a common ancestry considering their functional and organizational similarities (Krem and Cera (2002)). Analysis of both systems on the domain level would help to reveal the evolutionary histories of vertebrate blood coagulation and Arthropod hemolymph clotting cascades.

The evolution of blood coagulation cascade proteins is widely studied (Patthy (1985, 1990); Davidson et al (2003); Jiang and Doolittle (2003); Ponczek et al (2008, 2012, 2020); Doolittle (2009); Kimura et al (2009)). However, as the amount and the quality of genomic data are increasing, the resolution of the evolutionary studies is improving. In this study, we aim to show possible evolutionary trajectories of the fundamental blood coagulation cascade proteins using not only vertebrate proteomes but also invertebrate proteomes. We tried to shed light on the evolution of the blood coagulation cascade using domain alignments between vertebrate and invertebrate proteomes.

## Methods

### Dataset Creation

We selected 18 proteomes (Table 1) to be able to study the domain arrangements occurring in the blood coagulation cascade in detail. These proteomes were selected to represent most of the vertebrate clades ranging from jawless vertebrates to mammals. Proteomes of three species belonging to Cnidaria and Tunicata clades were also downloaded as outgroups from Ensembl (Hunt et al (2018)), NCBI (Johnson et al (2008)) and UniProt (UniProt Consortium (2018)) databases (Supplementary Table S1). Two Arthropoda species were also used to reveal domain similarities between blood coagulation and hemolymph clotting. The proteomes were cleared of isoforms and only the longest isoforms were retained. The isoformCleaner program from the dw-helper suite (https://zivgitlab.uni-muenster.de/domain-world/dw-helper) was used with settings ‘-r “gene[: =] \\s*([\\S] +)[\\s]*”’ for proteomes from Ensembl and the settings ‘-r “GN[: =] \\s*([\\S] +)[\\s]*”’ for proteomes from Uniprot. NCBI proteomes could not be handled with that program, so a python script (provided together with the study data) has been used. To detect domain arrangements, we used PfamScan (Finn et al (2014)) using Pfam database v32 (El-Gebali et al (2019)). The blood coagulation domains in each studied species are given in Table 1.

**Table 1:** Blood clotting domains in different species

		Species	Blood coagulation	Cu-oxidase	Cu-oxidase-2	Cu-oxidase-3	fn1	fn2	PAN_1	Gla	Thrombin light	VWA_N2
Chordata	Vertebrata	*Petromyzon marinus*	✓		✓	✓	✓	✓		✓	✓	
		*Eptatretus burgeri*	✓		✓	✓	✓	✓	✓	✓	✓	
		*Callorhinchus milii*	✓		✓	✓	✓	✓	✓	✓	✓	
		*Danio rerio*	✓		✓	✓	✓	✓	✓	✓	✓	
		*Latimeria chalumnae*	✓	✓	✓	✓	✓	✓	✓	✓	✓	✓
		*Xenopus tropicalis*	✓		✓	✓	✓	✓	✓	✓	✓	
		*Pelodiscus sinensis*	✓		✓	✓	✓	✓	✓	✓	✓	
		*Pseudonaja textilis*	✓		✓	✓	✓	✓	✓	✓	✓	
		*Crocodylus porosus*	✓		✓	✓	✓	✓	✓	✓	✓	
		*Gallus gallus*	✓	✓	✓	✓	✓	✓	✓	✓	✓	
		*Ornithorhynchus anatinus*	✓	✓	✓	✓	✓	✓	✓	✓	✓	✓
		*Vombatus ursinus*	✓	✓	✓	✓	✓	✓	✓	✓	✓	✓
		*Tursiops truncatus*	✓	✓	✓	✓	✓	✓	✓	✓	✓	✓
		*Physeter catodon*	✓	✓	✓	✓	✓	✓	✓	✓	✓	✓
		*Balaenoptera musculus*	✓	✓	✓	✓	✓	✓	✓	✓	✓	✓
		*Canis lupus familiaris*	✓	✓	✓	✓	✓	✓	✓	✓	✓	✓
		*Mus musculus*	✓	✓	✓	✓	✓	✓	✓	✓	✓	✓
		*Homo sapiens*	✓	✓	✓	✓	✓	✓	✓	✓	✓	✓
	Tu	*Ciona intestinalis*		✓	✓	✓	✓	✓	✓	✓		
		*Oikopleura dioica*		✓	✓	✓	✓	✓	✓	✓		
Cn		*Stylophora pistillata*		✓	✓	✓		✓	✓	✓		
Ar		*Drosophila melanogaster*		✓	✓	✓			✓			
		*Limulus polyphemus*							✓			

The domains C8, EGF, EGF_CA, FXa_inhibition, F5_F8_type_C, Kringle, Sushi, Transglut_core, Trypsin, VWA, VWC, VWD, Tranglut_C, Transglut_N appear in all species and are therefore not shown in this table

*Cn* Cnidaria, *Ar* Arthropoda, *Tu * Tunicata

### Identifying Blood Coagulation Genes

We used OrthoFinder (v2.3.12) to detect orthologous proteins between species (Emms and Kelly (2015)). We performed three OrthoFinder searches using studied proteomes: one for vertebrates to detect orthologues of blood coagulation proteins, one for arthropods including human, mouse and zebrafish proteomes as references, to detect orthologues of hemolymph clotting proteins and one for invertebrates including human, mouse and zebrafish as references, to detect orthologues of BLASTP target proteins for Prothrombin, FVII, FX. Exonerate (v2.2.0) (Slater and Birney (2005)) was run in ‘protein to genome’ mode to verify whether a certain ortholog was found when there were discrepancies between the current data and the literature.

### Identifying Potential Origins of Domains

One of the goals of this study was to identify potential origins of domains involved in the blood coagulation cascade. Specific databases were created containing only sequences from our data set belonging to one specific domain. To shed light on the domain evolution of the blood coagulation cascade, domain level reciprocal BLASTP (v2.9.0) (Altschul et al (1990)) searches were executed between these databases. The domains of blood coagulation cascade proteins in the most basal species were blasted against domains in the closest species not possessing the respective proteins.

If the query domain is found within the first three hits (when sorted by e-value) of the reciprocal search, the target is considered a good hit. To perform a BLASTP search, we first built a local database using all respective domains from a target proteome.

### Analysis of “missing” Genes

In a few cases, orthology analysis was not be able to identify some proteins in the studied species which should be present according to the current literature and in some cases orthology analysis identify some proteins which should not be present in the studied species. In both cases the same methodology was used: We used the Ensembl genome browser (Howe et al (2021)) to determine which genes are the closest on either side of the missing gene. We then checked those two genes in the target species to identify the potential region where the gene should be. Furthermore, we performed protein to genome exonerate searches to support the results. Additionally, to ensure that the gene is not only missing in our set of species, we also searched the entire clade surrounding that species for potential matches. We used NCBI blastp and tblastn together with the non-redundant databases (nr and nt). As the query we used a known ortholog from our species set that was most closely related to that clade. If all the analyses show clear signs of the absence of the gene (i.e., no synteny, bad exonerate and Blast hit(s)), we consider the gene is lost during the evolution of vertebrates. When the results were not clear, we label the gene as ambiguous.

### Phylogenetic Tree

MUSCLE (v5.1) was used to align blood coagulation cascade proteins in the proteomes (Edgar (2004)). We built phylogenetic trees using IQ-TREE (1.6.1) with 1000 bootstrapping runs (Minh et al (2020)). We, then, used FigTree (1.4.4) to visualize the phylogenetic trees (Rambaut (2014)). All software was used with default parameters following the manuals.

## Results and Discussion

### Conservation and Domain Arrangements of the Proteins

Putative orthologs of blood coagulation proteins were searched in the study species using OrthoFinder and BLASTP. Table 2 lists the presence and absence of the proteins in the given clades. While most of the data were consistent with the current literature, we could not identify one of the cofactors of the cascade, FV, in jawless vertebrates. It was suggested that FX exists in jawless vertebrates along with its cofactor FV (Doolittle (2009)). Even though FX is found in jawless vertebrates, FV could not be found by Orthofinder, BLASTP, or synteny analyses. However, Exonerate analysis yielded short fragments of alignment (see study data). FV, FVIII, hephaestin, and ceruloplasmin, which are multicopper oxidases, are very similar in their sequence (Vasin et al (2013)). Therefore, one of the multicopper oxidase protein family proteins might have been misidentified as FV using the NCBI Trace database (NCBI Trace Archive) which contains uncurated DNA sequences (see below: *Origin of Domains and Evolution of Domain Arrangements*).

**Table 2:** Absence and presence of coagulation cascade proteins in vertebrates

	V	VII	VIII	IX	X	XI	XII	XIIIA	XIIIB	C	Z	S	THRB	PLG	KLKB	vWF
Jawless vertebrate (*n* = 2)	**?**	+	−	−	+	−	−	−	−	−	−	+	+	+	−	+
Cartilaginous fish (*n* = 1)	+	+	+	+	+	−	−	+	−	**+**	**+**	+	+	+	−	+
Ray-finned fish (*n* = 1)	+	+	+	+	+	−	−	+	+	+	+	+	+	+	−	+
Lobe-finned fish (*n* = 1)	+	+	+	+	+	−	−	+	+	+	+	+	+	+	+	+
Amphibian (*n* = 1)	+	+	+	+	+	−	+	+	+	+	+	+	+	+	+	+
Reptile (n = 2)	+	+	+	+	+	−	+	+	+	+	+	+	+	+	+	+
Bird (*n* = 1)	+	+	+	+	+	−	−	+	+	+	+	+	+	+	+	+
Monotreme (*n* = 1)	+	+	+	+	+	+	+	+	+	+	+	+	+	+	+	+
Marsupial (*n* = 1)	+	+	+	+	+	+	+	+	+	+	+	+	+	+	+	+
Cetacean (*n* = 2)	+	+	+	+	+	+	−	+	+	+	+	+	+	+	−	+
Placental (*n* = 3)	+	+	+	+	+	+	+	+	+	+	+	+	+	+	+	+

Coagulation factors are depicted by using only their number (i.e., Coagulation factor V is depicted as V), S: Vitamin K-dependent protein S, THRB: Prothrombin, PLG: Plasminogen, KLKB: Plasma Kallikrein, vWF: von Willebrand factor. + , − , ? represent, presence, absence and unknown, respectively. Most of the data were found to be in agreement with current literature (Jiang and Doolittle (2003); Ponczek et al (2008, 2012, 2020); Doolittle (2009)). Data in disagreement with current literature or are new in this study are marked in bold

FXII is known to be missing in cetaceans (Robinson et al (1969); Huelsmann et al (2019); Ponczek et al (2020)). In the study presented here, a putative ortholog of FXII was identified in the bottlenose dolphin *Tursiops truncatus*. However, according to Exonerate results, the genomic region in T. truncates that spans most of the FXII protein has four nonsense mutations (Supplementary Figure S2). Exonerate runs on other cetaceans, namely sperm whale (*Physeter catodon*) and blue whale (*Balaenoptera musculus*), showed similar or worse alignments. Therefore, we confirmed that FXII is pseudogenized in cetaceans. According to previous studies, a pseudogene conversion by point mutations has led to this protein prediction (Semba et al (1998)). FXII is activated through inorganic molecules (e.g., soil). Cetaceans and birds have little to no contact to soil, therefore, it has been suggested that factor FXII has lost its importance and was subsequently lost (Juang et al (2020)).

**Fig. 1:**
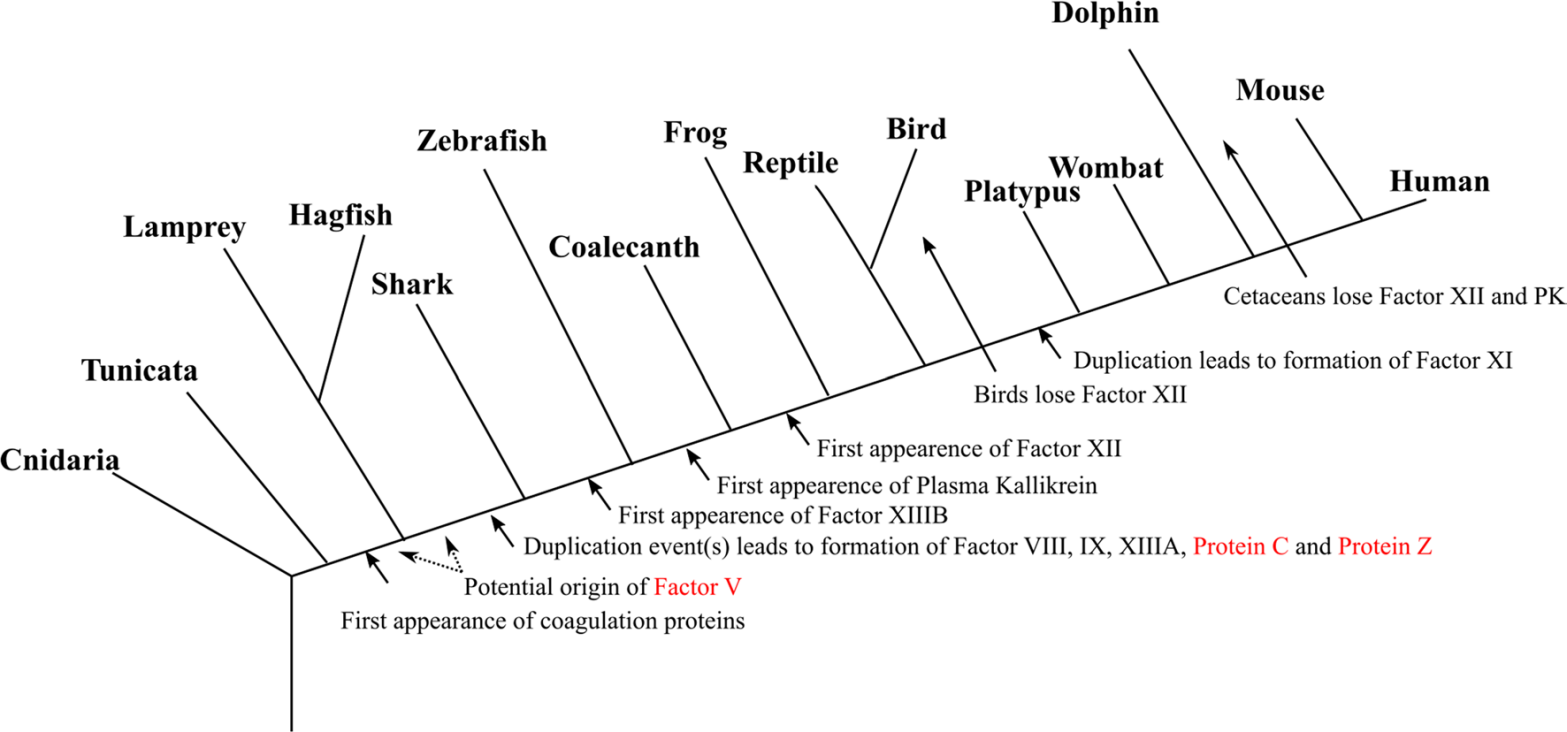
Appearance and disappearance of the studied coagulation proteins on the vertebrate phylogeny. The proteins marked in red show the updated and/or improved resolution compared to the literature. This figure is a modified version of a previous study by Doolittle (2009)

FXI and Plasma Kallikrein (PK) are paralogs that have the same domain arrangement: four tandem apple domains (*PAN 1*) followed by a *Trypsin* protease domain (Supplementary Table S2). Both PK and FXI are not present in jawless vertebrates, cartilaginous fish and ray-finned fish confirmed by exonerate, synteny and BLAST results. The last ancestral protein of PK and FXI should have appeared before the divergence of lobe-finned fish. It is found that, while PK is present in almost all tetrapods except for the cetaceans, a PK duplication before the divergence of mammals led to the emergence of FXI as discussed by Ponczek et al (2008, 2020). Figure 1 shows the emergence and loss of the studied blood coagulation proteins on the vertebrate evolutionary tree.

In terms of their domain arrangements, most of the blood coagulation cascade proteins are well conserved in all vertebrates. FIX, FX, Protein C, and Protein Z have identical domain arrangements (*Gla – EGF – Fxa inhibition – Trypsin*). Both, FXI and PK have the domain arrangement *PAN 1 – PAN 1 – PAN 1 – PAN 1 – Trypsin*. As for FXII, the domain arrangement is *fn2 – EGF – fn1 – EGF – Kringle – Trypsin*. Plasminogen has the domain arrangement *PAN 1 – Kringle – Kringle – Kringle – Kringle – Kringle – Trypsin*. Lastly, the domain arrangement of Prothrombin is *Gla – Kringle – Kringle – Thrombin light – Trypsin*. Even though serine proteases of the cascade are well conserved, cofactors are diverse in terms of the number of repetitions of the domains. FV had a variable number of LSPR domain repeats among different vertebrates, ranging from 24 in species *Balaenoptera musculus* to 38 in species *Physeter catodon,* while FVIII has a different number of domain repeats in *Copper oxidases* (Supplementary Table S3–4).

### Arthropod Hemolymph Clotting

We next investigated whether Arthropod hemolymph coagulation cascade proteins and vertebrate blood coagulation cascade proteins share a common ancestor. Unlike the blood coagulation cascade which is highly conserved among vertebrates, there are diverse proteins and cascades in different Arthropod clades. Therefore, we used hemolymph coagulation proteins from different members of Arthropoda to study this question.

The hemolymph coagulation cascade of *Limulus polyphemus* (Atlantic horseshoe crab), a non-insect Arthropod, was investigated as its coagulation proteins are widely studied. Hemolymph clotting in *L. polyphemus* is controlled by a proteolytic cascade similar to vertebrates. The cascade is controlled by coagulation factors B, C, and G, Proclotting enzyme, Coagulogen, Transglutaminase, and Coagulation inhibitors (Tanaka et al (1991); Theopold et al (2004); Schmid et al (2019)). Even though the cascade is similar to the vertebrate coagulation cascade in terms of proteolytic activity, proteins involved in the proteolytic cascade of *L. polyphemus* have completely different domain arrangements from the vertebrate coagulation cascade proteins. None of the blood coagulation cascade proteins share a single domain with these proteolytic proteins in *L. polyphemus* except for Trypsin, which is a common serine protease domain (Supplementary Table S5).

As for insect Arthropods, we used *Drosophila melanogaster* as a reference species. Hemolymph clotting in *D. melanogaster* is controlled by a small set of proteins: Fondue, Hemolectin, Transglutaminases, and Prophenoloxidases (Lindgren et al (2008); Wang et al (2010); Schmid et al (2019)) (Supplementary Table S6).

**Fig. 2:**
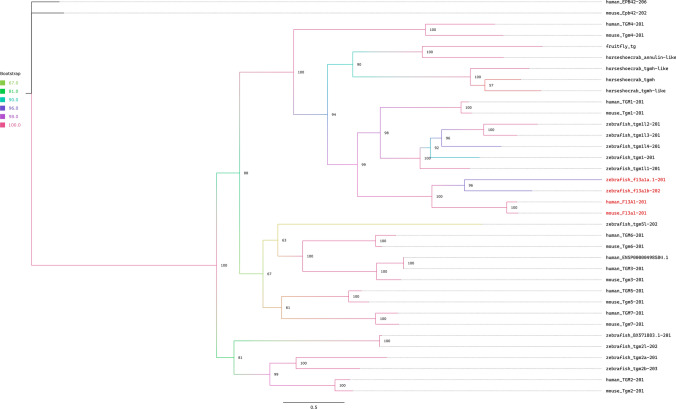
Phylogenetic tree with bootstrap values of zebrafish, human and mouse FXIIA, transglutaminases and arthropod transglutaminases. FXIIAs are depicted in red

The vertebrate and the invertebrate coagulation systems consist of different proteins with different domain arrangements. The only exception from this is the Transglutaminase in Arthropods, which has the same domain arrangement as FXIII A chain in vertebrates: *Transglut N – Transglut core – Transglut C – Transglut C*. Not only do they share the same domain organization, but they both play an important role on increasing the clotting efficacy. While FXIII A chain stabilizes fibrin clots (Byrnes et al (2015)), Transglutaminase in *Drosophila melanogaster* and *Limulus polyphemus* stabilizes clotting molecules proxin, stablin, and fondue (Lindgren et al (2008); Dushay (2009)). Therefore, we built a phylogenetic tree using all proteins from horseshoe crab, fruit fly, zebrafish, mouse and human which have the same domain arrangement as transglutaminases (Fig. 2). Similarity of domain arrangements are a potential indication for homology (Grassi et al (2010)). With high bootstrap values (> 94) and same domain arrangements, vertebrate FXIIIA and arthropod transglutaminases share a common ancestor.

**Fig. 3:**
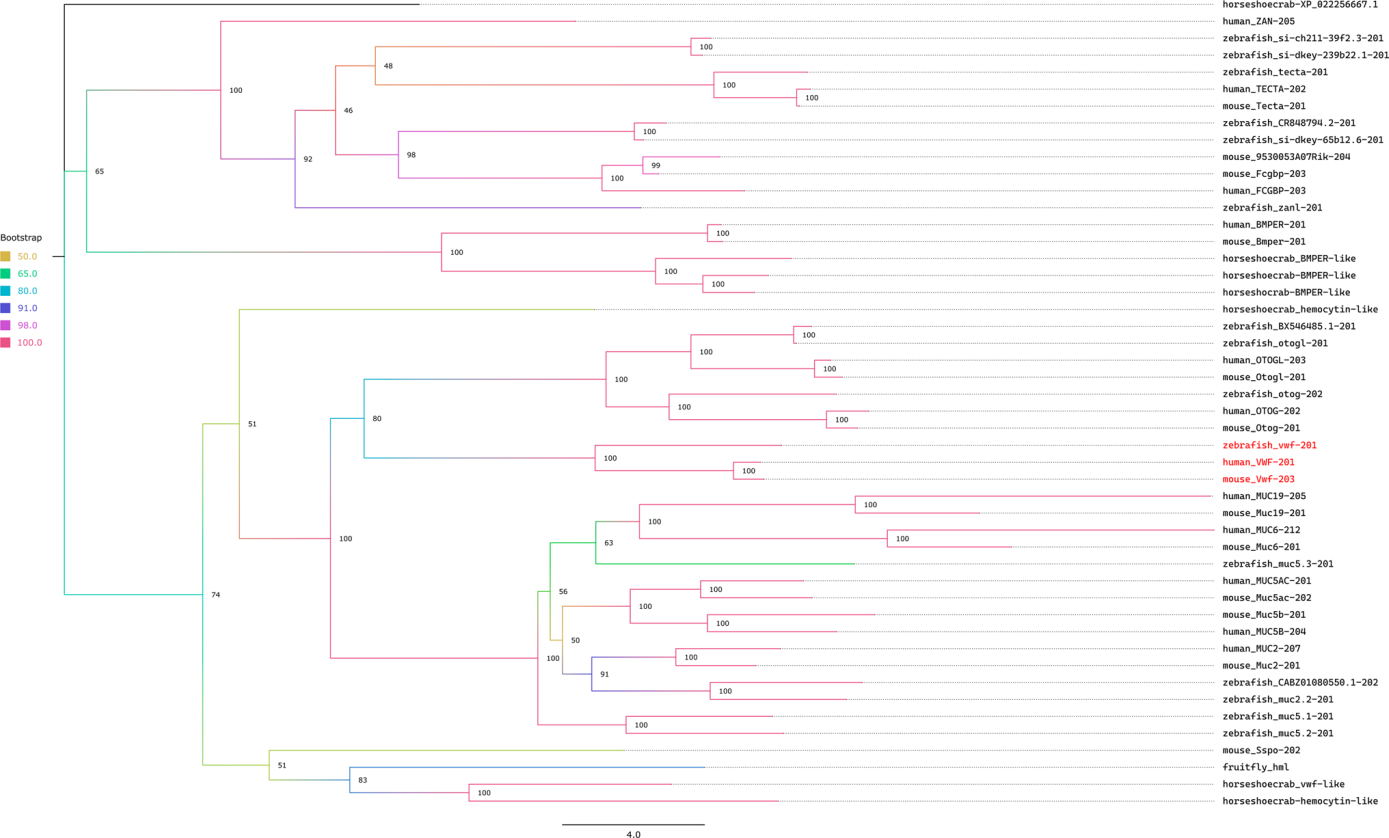
Phylogenetic tree with bootstrap values of proteins containing VWD, TIL and C8 domains in zebrafish, mouse, human, fruitfly, and horseshoe crab. vWFs are depicted in red

Besides the identical domain arrangements of coagulation FXIII subunit A and Transglutaminase, *D. melanogaster* hemolectin and von Willebrand factor share three domains: VWD, TIL and C8. Therefore, we built a phylogenetic tree of the proteins which have VWD, TIL and C8 domains with shared domains and similar modularity, it is highly possible that these two proteins share a common ancestry. The constructed phylogenetic tree with bootstrap values can be found in Fig. 3.

### Origin of Domains and Evolution of Domain Arrangements

**Fig. 4:**
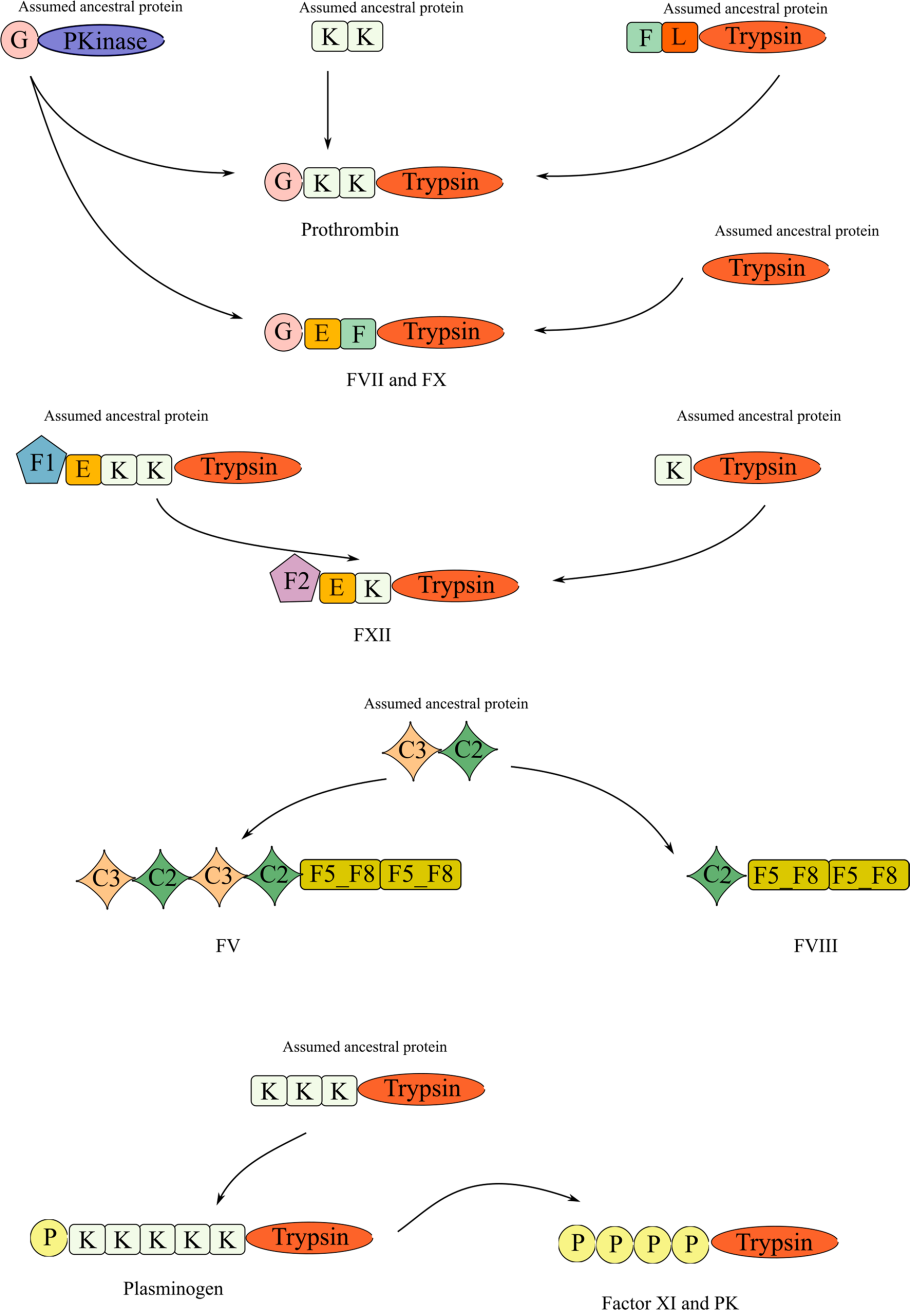
Proposed evolutionary trajectories of blood coagulation cascade proteins. (K: Kringle, P: PAN1, G: Gla, E: EGF, F: FXa_inhibition, L: Ldl_recept_b, F2: fn2, C2: Cupper oxidase 2, C3: Cupper oxidase 3, F5_F8: F5_F8 type C, PKinase: PKinase_Tyr)

Blood coagulation cascade proteins have similar domain arrangements indicating a possible common evolutionary history. Human coagulation factors FIX, FX, Protein C and Protein Z share the same domain arrangement (*Gla – EGF – Fxa inhibition – Trypsin*), while FVII has *Gla – EGF – Trypsin*. Besides the above arrangements, *Gla—EGF—Fxa inhibition* arrangement is also found within the different domain arrangement of Protein S (*Gla – EGF – Fxa inhibition – EGF CA – EGF CA – Laminin G 1 – Laminin G 2*). To reveal evolutionary histories of these domain arrangements, we performed reciprocal BLASTP searches using samples of FVII and FX from the most basal species. Unfortunately, reciprocal BLASTP searches on the domain level did not result in reciprocal matches in most of the cases. Next, we extended our search including the best three hits of the BLASTP results. BLASTP searches using *Petromyzon marinus* and *Eptatretus burgeri* FVII and FX domains against *Oikopleura dioica*, *Ciona intestinalis*, and *Stylophora pistillata* yielded two proteins in *O. dioica*: E4WZI2 and E4X518 (Fig. 4).

Protein S and FVII (and by extension FIX, FX, Protein C, Protein Z which have a similar domain arrangement as FVII) only differ in their terminal domains. While FVII has serine protease activity provided by its terminal *Trypsin* domain, Protein S has *EGF CA, Laminin G 1* and *Laminin G 2* domains in its terminal position. During the evolution of vertebrates, terminal domain loss and gain events may have led to the emergence of Protein S and FVII/FX. Since jawless vertebrates have only FVII and FX with this domain arrangement, all other serine proteases having this domain arrangement (FIX, Protein C, Protein Z) are likely the result of gene duplications. In humans, FVII, FX, and Protein Z are located close to each other on the forward strand of 13th chromosome (2–9 kbp between each of them) which supports the role of gene duplication in the emergence of these serine proteases.

The same strategy was applied to the domains of Prothrombin and FXII. As for Prothrombin, two proteins from Tunicata, ENSCINP00000019224 and E4WTL6 and one protein from Cnidaria, A0A2B4SCC6, were found to be the best hits for each domain, *Gla, Kringle* and *Trypsin*, respectively (Fig. 4). The best hit for the Gla domain, ENSCINP00000019224, was found to be orthologous to the best hit of FVII Gla, E4WZI2, indicating a common origin. As for FXII, we performed reciprocal BLASTP searches between a frog (*X. tropicalis*), our most basal species with FXII, and the lobe-finned fish (*L. chalumnae*). Two hits were found for *Kringle* and *Trypsin* domains, ENSLACP00000015011 (tissue type plasminogen activator) and ENSLACP00000010869 (urokinase plasminogen activator), respectively. It is proposed that FXII arose from duplication of Hepatocyte Growth Factor Activator (HGFAC) (Ponczek et al (2008, 2020)), even though both proteins share almost identical domain arrangements. Considering reciprocal domain matches between frog FXII and coelacanth plasminogen activators, FXII emerged as a result of a duplication event around ∼400 mya after the divergence of tetrapods.

**Fig. 5:**
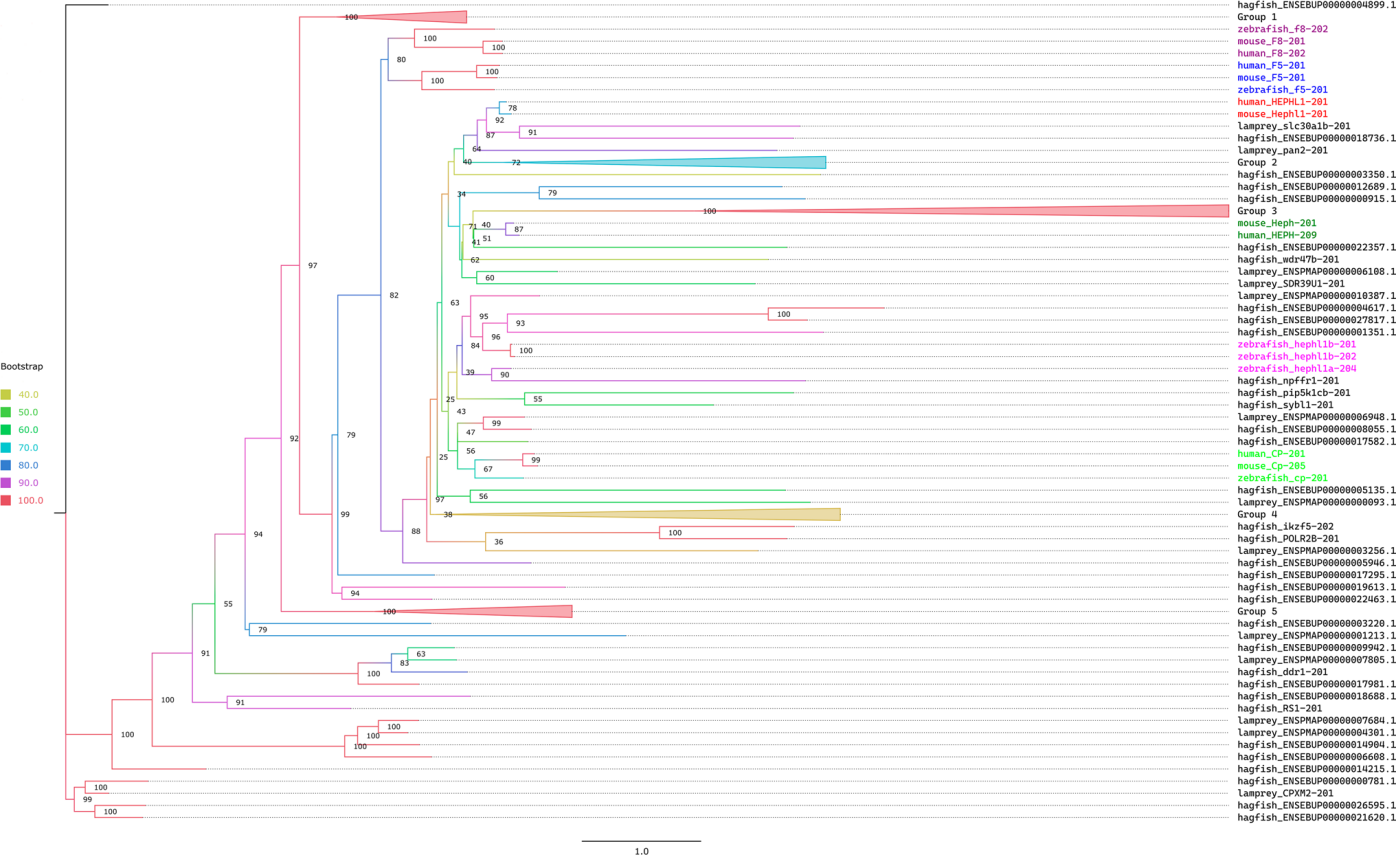
Phylogenetic tree with bootstrap values of FV, FVIII, Ceruloplasmin and Hephaestin from zebrafish, mouse and human together with putative orthologs of FV in jawless vertebrates. Some branches (Groups 1–5) containing only jawless vertebrate proteins were collapsed to increase readability. For full tree, see supplementary Figure S4

As for FV and FVIII, it was suggested that a single copy of FV is present and FVIII missing in jawless vertebrates (Doolittle (2009)). However, the identification of FV might be a misidentification of a hephaestin/ceruloplasmin-like protein. Reciprocal BLASTP searches using FV, FVIII, Ceruloplasmin and Hephaestin from *Danio rerio, Callorhinchus milii*, and *Homo sapiens* as queries against *Petromyzon marinus* and *Eptatretus burgeri* proteomes showed that there is not a reciprocally “perfect match” between FV and any jawless vertebrate protein. Hephaestin has a reciprocal match in the lamprey proteome which is also the best hit for FV. Therefore, this lamprey protein is most likely an ortholog of Hephaestin instead of FV. To further support this finding, we built a gene tree using all possible putative orthologs of FV found by BLASTP (71 candidates for *E. burgeri* and 31 candidates for *P. marinus*), together with *H. sapiens, D. rerio*, and *C. milii* FV, FVIII, hephaestin, and ceruloplasmin. While FV and FVIII from shark, zebrafish and human are clustered together, hephaestins and other jawless vertebrate proteins are found in different clades (Fig. 5). Besides, NCBI BLASTP and TBLASTN searches using human FV against jawless vertebrates yielded *P. marinus* hephaestin-like protein. To further support, we investigated synteny using the genomic region of FV in *D. rerio* and *C. milii*. In both species, FV is found between the genes ccdc80 and gpr161. However, the orthologs of ccdc80 and gpr161 are found in two different scaffolds in jawless vertebrates (FYBX02010127.1 and FYBX02010267.1 in hagfish and GL476438 and GL480261 in lamprey, respectively). (Supplementary Figure S3). Lastly, we performed protein to genome Exonerate runs using human FV against lamprey and hagfish genomes. Even though there is no good alignment that spans most of the FV protein, there are short sequences in jawless vertebrates that align to smaller fragments of FV (see study data). Searches on the current genomic data of jawless vertebrates, do not yield a concrete result. Considering all these analyses, FV is therefore potentially not present in jawless vertebrates but rather emerged in Gnathostomata after the split from Cyclostomata around ∼600 mya. However, since the available jawless vertebrate genomes are in low quality, this finding could be an artifact of the fragmented genomes.

As for von Willebrand factor, tracing the domain arrangement evolution of the protein is more difficult due to its modular structure. Similar to FV and FVIII, von Willebrand factor is also composed of a highly modular domain arrangement, making it similar to Arthropod Hemolectin (Supplementary Table S2, S6). In all studied animal groups, there are similar modular *TIL, VWD*, and *C8* containing proteins. The phylogenetic tree of *TIL, VWD*, and *C8* domains-containing proteins from human, mouse, zebrafish, horseshoe crab, and fruitfly shows homology between vertebrate von Willebrand factor and Arthropod hemolectin. However, to gain a deeper understanding of the evolution of von Willebrand factor and Hemolectin, future studies should include animals deeper in the phylogeny and fungi species, *Fusarium sp.* and *Aspergillus sp.*, as their proteomes contain more ancestral versions of the aforementioned domains.

Unfortunately, due to the long evolutionary time frame of over ∼600 mya and low sequence similarity, it is difficult to determine possible evolutionary trajectories of blood coagulation cascade protein domains. However, our work here increases the resolution of Doolittle’s work on vertebrate blood coagulation cascade evolution (Doolittle (2009)).

## Conclusion

The blood coagulation cascade is very complex and, although some proteins are missing in early vertebrates, the coagulation proteins are structurally well conserved among all vertebrates. While Arthropod hemolymph clotting seems functionally similar to the blood coagulation cascade, only two proteins in the blood coagulation cascade (FXIII subunit A and von Willebrand factor (vWF)) have similar domain arrangements to hemolymph clotting proteins Transglutaminase and Hemolectin, respectively. The remainder of the blood coagulation proteins, including all the key proteins, are found to have different evolutionary histories compared to Arthropod hemolymph clotting proteins.

Here, not only do we show new data on the emergence of FXIII, Protein C and Protein Z, but we also propose possible evolutionary trajectories for vertebrate coagulation factors which were derived from domain similarity assessments. The first appearance of FV could not be exactly defined. Although a previous publication (Doolittle et al (2008)) showed a potential existence in lamprey, our own analyses in hagfish and lamprey showed no clear candidate in the available jawless genomes. We confirm the origins of the blood coagulation proteins in Tunicata and Cnidaria and describe the evolution of the blood coagulation cascade at various levels: Jawless to jawed vertebrates and teleosts to tetrapods. Analyses on domain re-arrangements suggest that gene and/or genome duplications during the evolution of the vertebrates led to the modern-day coagulation cascade proteins.

## Supplementary Information

Below is the link to the electronic supplementary material.

Supplementary file1 (PDF 313 kb)

## Data Availability

Data belonging to this study (e.g., proteomes, BLASTP results) have been uploaded to zenodo.org: 10.5281/zenodo.6576868
