# Supplementary material for: Domain Evolution of Vertebrate Blood Coagulation Cascade Proteins
Source: J Mol Evol. 2022 Oct 1;90(6):418–28. doi: 10.1007/s00239-022-10071-3 (PMC9643190; doi:10.1007/s00239-022-10071-3)
Supplement: Supplementary file 1 — Supplementary file1 (PDF 313 kb) [file 239_2022_10071_MOESM1_ESM.pdf]

Domain Evolution of Vertebrate Blood Coagulation Cascade Proteins

Abdulbaki Coban, Erich Bornberg-Bauer and CarstenKemena

Institute for Evolutionary Biology, WWU Münster, Münster,Germany

Corresponding author(s). E-mail(s): c.kemena@wwu.de

Table S1: Studied species and the source of proteomes

Order		Proteome	Source
Chordata	Vertebrata	*Petromyzon marinus*	Ensembl Release 105
		Eptatretus burger	
		Callorhincus milii	
		Danio rerio	
		Latimeria chalumnae	
		Xenopus tropicalis	
		Pelodiscus sinensis	
		Pseudonaja textilis	
		Crocodylus porosus	
		Gallus gallus	
		Ornithorhynchus anatinus	
		Vombatus ursinus	
		Tursiops truncates	
		Physeter catodon	
		Balaenoptera musculus	
		Canis lupus familiaris	
		Mus musculus	
		Homo sapiens	
	Tunicata	*Ciona intestinalis*	
		Oikopleura dioica	Uniprot: UP000001307
Cnidaria		*Stylophora pistillata*	Uniprot: UP000225706
Arthropoda		*Drosophila melanogaster*	Ensembl Release 105
		Limulus polyphemus	NCBI SAMN02953878

Table S2: Human coagulation factors with their respective domain arrangements

**Protein Name**	**Domain Arrangement**
Factor V	Cu_oxidase_3(x2) – F5_F8_type_C(x2)
Factor VII	Gla – EGF – Trypsin
Factor VIII	Cu_oxidase_3 – Cu_oxidase – Cu_oxidase_3 – Cu_oxidase_2 – F5_F8_type_C(x2)
Factor IX	Gla – EGF – FXa_inhibition – Trypsin
Factor X	Gla – EGF – FXa_inhibition – Trypsin
Factor XI	PAN_1(x4) – Trypsin
Factor XII	fn2 – EGF – fn1 – EGF – Kringle – Trypsin
Factor XIIIA	Transglut_N – Transclut_core – Transclut_C – Transglut_C
Factor XIIIB	Sushi(x8)
Plasma Kallikrein	P450 - PAN_1(x4) – Trypsin
Plasminogen	PAN_1 – Kringle(x5) – Trypsin
Prothrombin	Gla – Kringle – Kringle – Thrombin_light – Trypsin
Protein C	Gla – EGF – FXa_inhibition – Trypsin
Protein S	Gla – EGF – Fxa_inhibition – EGF_CA(x2) – Laminin_G_1 – Laminin_G_2
Protein Z	Gla – EGF – FXa_inhibition – Trypsin
vWF	VWD – C8 – TIL– VWD – C8 – TIL (x2) – VWD – C8 – TIL – VWA_N2 – VWA (x3) – VWD – C8 – VWC (x3)

Table S3: Coagulation Factor V domain arrangements in studied vertebrate species

Protein Name	Domain Arrangement
ENSBMSP00010012361	Cu_oxidase_3 - LSPR(x24) – Cu_oxidase_3 - F5_F8_type_C(x2)
ENSCAFP00845003315	Cu_oxidase_3 - LSPR(x34) – Cu_oxidase_3 - F5_F8_type_C(x2)
ENSCMIP00000027689	Cu_oxidase_3 – Cu_oxidase_2 – Cu_oxidase_3 – Cu_oxidase_2 – F5_F8_type_C(x2)
ENSCPRP00005005764	Cu_oxidase_3 – Cu_oxidase_3 – F5_F8_type_C(x2)
ENSDARP00000072528	Cu_oxidase_3 – Cu_oxidase_3 – Cu_oxidase_3 – F5_F8_type_C(x2)
ENSGALP00000047983	Cu_oxidase_3 – Cu_oxidase_3 – Cu_oxidase_3 – Cu-oxidase_2 – F5_F8_type_C(x2)
ENSP00000356770	Cu_oxidase_3 – Cu_oxidase_3 – F5_F8_type_C(x2)
ENSLACP00000018437	Cu_oxidase_3 – Cu_oxidase_3 – Cu_oxidase_3 – Cu-oxidase_2 – F5_F8_type_C(x2)
ENSMUSP00000083204	Cu_oxidase_3 – Cu_oxidase_3 – Cu_oxidase_3 – F5_F8_type_C(x2)
ENSOANP00000001346	Cu_oxidase_3 – Cu_oxidase_3 – F5_F8_type_C(x2)
ENSPCTP00005005300	Cu_oxidase_3 - LSPR(x38) – Cu_oxidase_3 - F5_F8_type_C(x2)
ENSPSIP00000002236	Cu_oxidase_3 – Cu_oxidase_3 – Cu_oxidase_3 – Cu-oxidase_2 – F5_F8_type_C(x2)
ENSPTXP00000023606	Cu_oxidase_3 – Cu_oxidase_3
ENSTTRP00000003817	Cu_oxidase_3 - LSPR(x28) – F5_F8_type_C(x2)
ENSVURP00010005461	Cu_oxidase_3 – Cu_oxidase_3 – F5_F8_type_C(x2)
ENSXETP00000005164	Cu_oxidase_3 – Cu_oxidase_3 – Cu_oxidase_3 – Cu-oxidase_2 – F5_F8_type_C(x2)

Table S4: Coagulation Factor VIII domain arrangements in studied vertebrate species

Protein Name	Domain Arrangement
ENSBMSP00010029765	Cu_oxidase_3 – Cu-oxidase – Cu_oxidase_3 – Cu_oxidase_2 – F5_F8_type_C(x2)
ENSCAFP00845041989	Cu_oxidase_3 – Cu-oxidase – Cu_oxidase_3 – Cu_oxidase_2 – F5_F8_type_C(x2)
ENSCMIP00000007556	Cu_oxidase_3 – Cu_oxidase_3 – F5_F8_type_C(x2)
ENSCPRP00005000078	Cu_oxidase_3 – Cu_oxidase_2 – F5_F8_type_C(x2)
ENSDARP00000130264	Cu_oxidase_2 – F5_F8_type_C(x2)
ENSGALP00000044539	Cu_oxidase_3 – Cu-oxidase_2 – F5_F8_type_C(x2)
ENSP00000353393	Cu_oxidase_3 – Cu-oxidase – Cu_oxidase_3 – Cu_oxidase_2 – F5_F8_type_C(x2)
ENSLACP00000016108	Cu_oxidase_3 – Cu_oxidase – Cu-oxidase_2 – F5_F8_type_C(x2)
ENSMUSP00000033539	Cu_oxidase_3 – Cu_oxidase_3 – Cu_oxidase_2 – F5_F8_type_C(x2)
ENSOANP00000043671	Cu_oxidase_3 – Cu_oxidase – Cu_oxidase_2 – F5_F8_type_C(x2)
ENSPCTP00005021866	Cu_oxidase_3 – Cu-oxidase – Cu_oxidase_3 – Cu_oxidase_2 – F5_F8_type_C(x2)
ENSPSIP00000019185	Cu_oxidase_3 – Cu-oxidase_2 – F5_F8_type_C(x2)
ENSPTXP00000006275	Cu_oxidase_3 – Cu_oxidase_2
ENSTTRP00000007713	Cu_oxidase_3 – Cu-oxidase – Cu_oxidase_3 – Cu_oxidase_2 – F5_F8_type_C(x2)
ENSVURP00010025397	Cu_oxidase_3 – Cu-oxidase – Cu_oxidase_3 – Cu_oxidase_2 – Cu_oxidase_2 - F5_F8_type_C(x2)
ENSXETP00000005164	Cu_oxidase_3 – Cu_oxidase_3 – Cu_oxidase_3 – Cu-oxidase_2 – F5_F8_type_C(x2)

Table S5: Horseshoe crab coagulation factors with their respective domain arrangements

**Protein Name**	**Domain Arrangement**
Coagulogen	Coagulin
Factor B	Trypsin
Factor C	LCCL – Lectin_C
Factor G alpha	Glyco_hydro_16 – RicinB_lectin2 (x2) – CBM_6 (x2)
Factor G beta	Trypsin
Intracellular coagulation inhibitor (1,2,3)	Serpin
Proclotting Enzyme	CLIP – Trypsin
Transglutaminase	Transglut_N – Transglut_core – Transglut_C – Transglut_C

Table S6: *Drosophila* coagulation factors with their respective domain arrangements

**Protein Name**	**Domain Arrangement**
Hemolectin	VWD – C8 – TIL – VWD – C8 – TIL (x2) – VWD – C8 – F5_F8_type_C (x2) – VWD – C8 – TIL – VWD – C8 – TIL
PPO1-2-3	Hemocynanin_M – Hemocynain_N – Hemocyanin_C
Transglutaminase	Transglut_N – Transglut_core – Transglut_C – Transglut_C

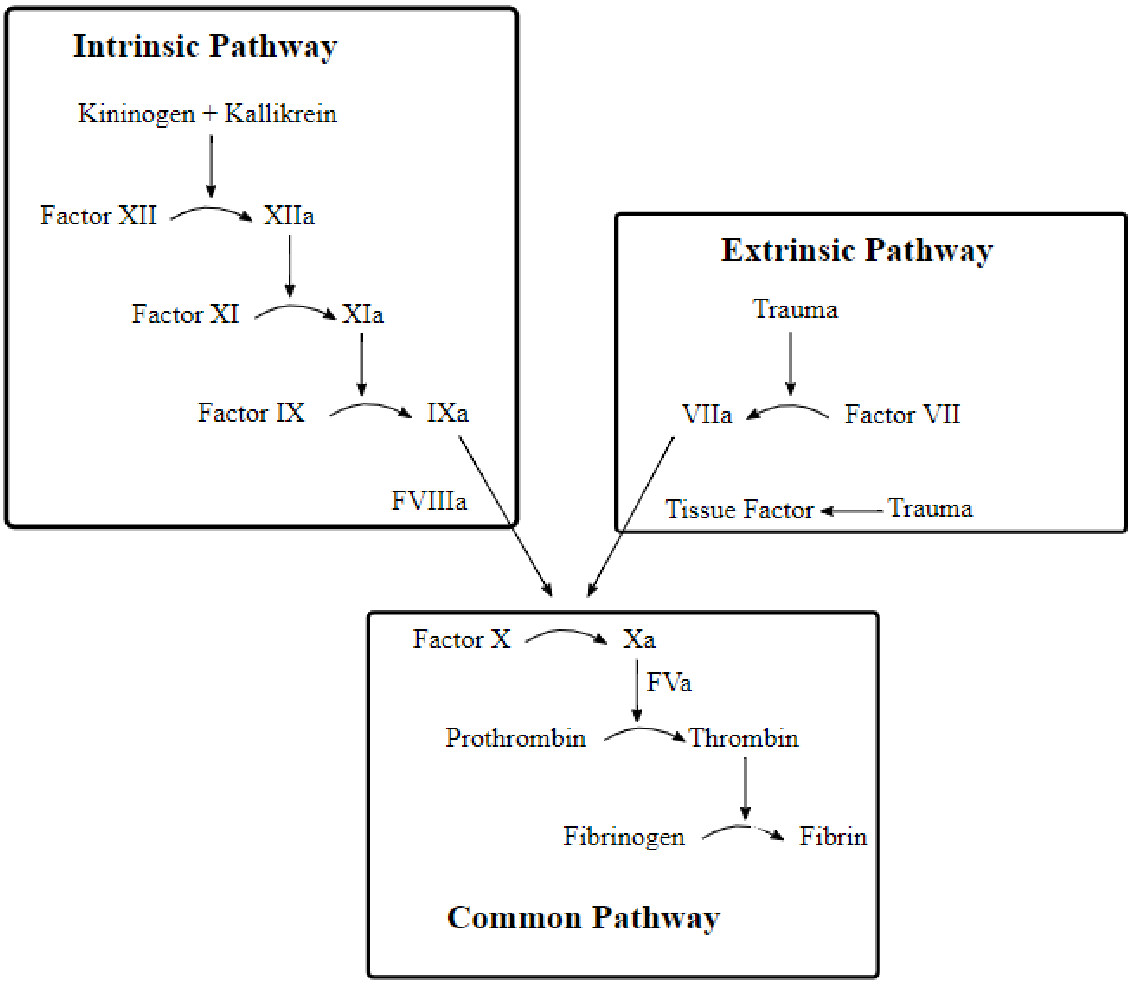

Figure S1: Intrinsic, extrinsic, and common pathways of blood coagulation

C4 Alignment:

Query: ENSP00000253496.3 pep chromosome:GRCh38:5:177402141:177409564:-1 gene:ENSG00000131187.10 transcript:ENST00000253496.4 gene_biotype:protein_coding transcript_biotype:protein_coding gene_symbol:F12 description:coagulation factor XII [Source:HGNC Symbol;Acc:HGNC:3530]

Target: GeneScaffold_2447 dna_sm:genescaffold genescaffold:turTru1:GeneScaffold_2447:1:316058:1 REF:[revcomp]

Model: protein2genome:local

Raw score: 1981

Query range: 0 -> 615

Target range: 229166 -> 215645

```
     1 : MetArgAlaLeuLeuLeuLeuGlyPheLeuLeuValSerLeuGluSerThrLeuSer  :     20
         |||||||||||||||||||||| !!||||||||||||||||||||.!!:!!! !
         MetArgAlaLeuLeuLeuLeuGlyValLeuLeuValSerLeuGluSerAlaValLeu+
229166 : ATGAGGGCTCTGCTGCTCCTGGGGGTCCTGCTGGTGAGCCTGGAGTCAGCAGTTTTGg : 229109

    21 :  >>>> Target Intron 1 >>>>  IleProProTrpGluAlaProLysGluHis :     29
                  366 bp           ! !|||||||||:!!||||||! !:!!  !
         +                         ++ThrProProTrpLysAlaProMetGlnSer
229108 : t.........................agACTCCACCTTGGAAAGCCCCCATGCAGAGC : 228716

    30 : LysTyrLysAlaGluGluHisThrVal{V}  >>>> Target Intron 2 >>>>  :     39
         !!.   :!!!.!..!  !.!.  !  !{ }           8651 bp
         AsnArgGluGlyArgMetGluGluCys{P}++                          +
228715 : AACAGGGAAGGAAGGATGGAGGAATGC{t}gt.........................a : 220036

    40 :  {al}LeuThrValThrGlyGluProCysHisPheProPheGlnTyrHisArgGlnLe :     57
          {!!}||||||||||||||||||||||||||||||||||||||||||||| !!|||||
         +{he}LeuThrValThrGlyGluProCysHisPheProPheGlnTyrHisTrpGlnLe
220035 : g{TT}CTCACTGTCACTGGGGAGCCCTGCCACTTCCCCTTTCAGTACCACTGGCAGCT : 219981

    58 : uTyrHisLysCysThrHisLysGlyArgProGlyProGlnPro{T}  >>>> Targe :     72
         ||||||||||! !! !|||!:!||| !!||||||||||.!.|||{ }
         uTyrHisLysPheIleHisArgGlyTrpProGlyProAspPro{G}++
219980 : GTATCACAAATTTATCCACAGAGGCTGGCCTGGCCCCGACCCT{G}gt.......... : 219933

    73 : t Intron 3 >>>>  {rp}CysAlaThrThrProAsnPheAspGlnAspGlnArg- :     85
```

```
        708 bp           {!!}|||||||||||||||||||||| ! :!!||||||||||#
                     --{ly}CysAlaThrThrProAsnPheLysLysAspGlnArg#
219932 : ..............ca{GG}TGTGCTACCACCCCCAACTTCAAGAAGGATCAGCGAT : 219189

    86 : -TrpGlyTyrCysLeuGluProLysLysValLys{A}  >>>> Target Intron  :     96
        #|||!.!|||||||||||||||||||||||||||||{|}            283 bp
        #TrpAlaTyrCysLeuGluProLysLysValLys{A}++
219188 : GTGGGCATACTGCCTGGAGCCAAAGAAAGTGAAA{G}gt.................. : 219151

    97 : 4 >>>>  {sp}HisCysSerLysHisSerProCysGlnLysGlyGlyThrCysValA :    112
               {||}|||||||||||||||!.!|||||||||||||||! !||||||||||
             ++{sp}HisCysSerLysHisLysProCysGlnLysGlyGluThrCysValA
219150 : ......ag{AT}CACTGCAGCAAACACAAACCCTGCCAGAAAGGAGAGACCTGTGTGA : 218823

   113 : snMetProSerGlyProHisCysLeuCysProGlnHisLeuThrGlyAsnHisCysGl :    131
        ||||||||!:!|||||||||||||:!!||||||.!.|||!!!||||||!!.||| !!||
        snMetProAsnGlyProHisCysIleCysProAspHisPheThrGlyLysHisGlyGl
218822 : ACATGCCAAATGGCCCACACTGCATCTGTCCAGATCACTTCACTGGGAAGCACGGCCA : 218766

   132 : nLys{G}  >>>> Target Intron 5 >>>>  {lu}LysCysPheGluProGln :    139
        |!:!{|}           139 bp           {||}|||||||||||||||||
        nArg{G}++                         ++{lu}LysCysPheGluProGln
218765 : GAGA{G}gt........................ag{AG}AAGTGCTTTGAGCCCCAG : 218603

   140 : LeuLeuArgPhePheHisLysAsnGluIleTrpTyrArgThrGluGlnAlaAlaValA :    159
        |||||| !!|||.!!!!.:!!||||||||||||:!!|||  !|||! !|||!.!||||
        LeuLeuTrpPheLeuGlnGluAsnGluIleTrpHisArgLeuGluLeuAlaGlyValA
218602 : CTTCTTTGGTTCCTCCAGGAGAATGAAATATGGCACAGGCTTGAGCTGGCAGGTGTAG : 218543

   160 : laArgCysGlnCysLysGlyProAspAlaHisCysGlnArgLeuAlaSerGln{A}   :    177
        ||!:!|||||||||!!.||||||:!!|||!!.|||:!!! !||||||||||||{!}
        laLysCysGlnCysAsnGlyProAsnAlaGlnCysLysProLeuAlaSerGln{V}++
218542 : CCAAGTGCCAGTGCAATGGTCCGAATGCCCAGTGCAAGCCACTGGCCAGCCAG{G}gt : 218488

   178 : >>>> Target Intron 6 >>>>  {la}CysArgThrAsnProCysLeuHisGly :    186
                161 bp           {.!}|||||||||||||||||||||:!!|||
                              ++{al}CysArgThrAsnProCysLeuAsnGly
218487 : ........................ag{TC}TGCCGTACCAACCCATGTCTCAATGGG : 218301
```

```
   187 : GlyArgCysLeuGluValGluGlyHisArgLeuCysHisCysProValGlyTyrThrG :    206
         |||!!!||||||:!!!.!||||||||||||||||||  !! !|||..!||||||.!!|
         GlySerCysLeuGlnAlaGluGlyHisArgLeuCysSerTyrProThrGlyTyrAlaG
218300 : GGCAGCTGCCTACAGGCGGAGGGCCACCGCCTGTGCAGTTACCCCACGGGCTACGCTG : 218241

   207 : lyAlaPheCysAspVal{A}  >>>> Target Intron 7 >>>>  {sp}ThrLy :    214
         ||  !!!.|||!!:|||{|}            146 bp           {||}  !||
         lyArgLeuCysGluVal{A}++                         ++{sp}HisLy
218240 : GACGCTTGTGCGAAGTG{G}gt.........................ag{AC}CACAA : 218071

   215 : sAlaSerCysTyrAspGlyArg<-><->GlyLeuSerTyrArgGlyLeuAlaArgThr :    231
         ||||!:!||||||||||! !|||      ||||||||||||||||||:!!||||||||||
         sAlaAsnCysTyrAspAspArgAspHisGlyLeuSerTyrArgGlyValAlaArgThr
218070 : GGCGAACTGTTACGACGACCGCGACCACGGGCTCAGCTACCGCGGCGTGGCCCGGACT : 218014

   232 : ThrLeuSerGlyAlaProCysGlnProTrpAlaSerGluAlaThrTyrArgAsnValT :    251
         ! !|||!!!||||||||||||||||||||||||||||||||||||||||| !!||||||||
         LysLeuTrpGlyAlaProCysGlnProTrpAlaSerGluAlaThrTyrTrpAsnValT
218013 : AAGCTCTGGGGTGCACCCTGTCAGCCGTGGGCCTCCGAGGCCACCTACTGGAATGTGA : 217954

   252 : hrAlaGluGlnAlaArgAsnTrpGlyLeuGlyGlyHisAlaPheCys{Ar}  >>>>  :    267
         ||||||||||||||! !||| !! !!|||||||! !|||||||||||||{||}
         hrAlaGluGlnAlaLeuAsnGlyArgLeuGlyAspHisAlaPheCys{Ar}++
217953 : CCGCAGAGCAAGCGCTGAACGGGAGACTGGGCGACCACGCCTTCTGC{CG}gt..... : 217902

   268 : Target Intron 8 >>>>  {g}AsnProAspAsnAspIleArgProTrpCysPhe :    278
            85 bp          {|}|||! !||||||||||! !||||||||||||||||
                         ++{g}AsnGlnAspAsnAspThrArgProTrpCysPhe
217901 : ...................ag{G}AACCAGGACAACGACACCCGCCCGTGGTGCTTT : 217788

   279 : ValLeuAsnArgAspArgLeuSerTrpGluTyrCysAspLeuAlaGlnCysGlnThrP :    298
         |||! !!..  !|||||||||||||! !.!.||||||  !|||||||! !|||||||.!!|
         ValTrpArgAspAspArgLeuSerSerAsnTyrCysArgLeuAlaLeuCysGlnAlaP
217787 : GTTTGGAGAGACGACCGACTAAGCTCGAATTATTGCCGCCTGGCACTGTGCCAGGCCC : 217728

   299 : roThr<-><-><-><->GlnAlaAlaProProThrProValSerProArgLeuHisVa :    313
         ||.!!            |||  ! !!|||||||! !! !:!!||| !!..!! !!!.
```

```
        roAlaProThrThrProGlnIleProProProIleArgIleSerSerGluHisGlnTy
217727 : CAGCCCCGACGACGCCCCAAATCCCTCCTCCGATCCGGATCTCATCTGAGCACCAGTA : 217671

   314 : lProLeuMetProAlaGlnProAlaProProLysProGlnProThrThrArgThrPro :    332
         !  !! !:!!|||:!!!   !!|||  !! !||||||||| !!|||||||!:!||||||
         rPheProLeuProSerLeuSerAlaLeuGlnLysProGlnSerThrThrGlnThrPro
217670 : CTTCCCTTTGCCCTCGCTTTCGGCTTTGCAGAAACCTCAGTCCACGACCCAGACCCCG : 217614

   333 : ProGlnSerGlnThrProGly{A}  >>>> Target Intron 9 >>>>  {la}L :    341
         ! !!:! !!! !||| !!|||{.}           97 bp            {.!}!
         LeuArgProLeuThrSerGly{C}++                        ++{ys}T
217613 : CTTCGACCCCTGACCTCAGGT{T}gt.........................ag{GC}T : 217490

   342 : euProAlaLysArgGluGlnProProSerLeuThrArgAsnGlyProLeuSerCysGl :    360
          !  !:!!  !! !|||||||! ! !! !!||| !!!!!   ||||||  !.!!|||||
         rpCysSerProProGluGlnArgThrProLeuProSerAlaGlyProAlaGlyCysGl
217489 : GGTGCTCGCCGCCCGAGCAGCGGACTCCCCTGCCCAGCGCGGGCCCGGCGGGCTGTGG : 217433

   361 : yGlnArgLeuArgLysSerLeuSerSerMetThrArgValValGlyGlyLeuValAla :    379
         |!:! !!||||!.!||| !!||||||||||:!!!:!||||||||||||||||||:!!|||
         yArgTrpLeuHisLysArgLeuSerSerLeuSerArgValValGlyGlyLeuMetAla
217432 : ACGGTGGCTCCACAAACGGCTGTCCTCGCTGAGCCGCGTCGTCGGGGGACTGATGGCG : 217376

   380 : LeuArgGlyAlaHisProTyrIleAlaAlaLeuTyrTrpGlyHisSerPheCysAlaG :    399
         |||! !|||||||||||||||||||||||||||!!   !|||!!.!:!||||||.!!|
         LeuProGlyAlaHisProTyrIleAlaAlaLeu***GlnGlyGlnAsnPheCysThrG
217375 : CTCCCCGGGGCGCACCCCTACATCGCCGCGCTGTAACAGGGCCAAAATTTCTGCACCG : 217316

   400 : lySerLeuIleAlaProCysTrpValLeuThrAlaAlaHisCysLeuGlnAsp{Ar}  :    417
         ||||||||||||!.!|||||||!! ||||||||||||||||||||||||||||:!!{||}
         lySerLeuIleValProCys***ValLeuThrAlaAlaHisCysLeuGlnAsn{Ar}+
217315 : GCAGCCTCATCGTCCCCTGTTGAGTGCTGACCGCGGCTCACTGCCTGCAGAAC{CG}g : 217260

   418 :  >>>> Target Intron 10 >>>>  {g}ProAlaProGluAspLeuThrValVa :    426
                 262 bp            {|}||||||||||||!!:|||||||||||
         -                       ++{g}ProAlaProGluGluLeuThrValVa
217259 : c.........................ag{A}CCTGCGCCAGAGGAGCTGACAGTGGT : 216973
```

```
   427 : lLeuGlyGlnGluArgArgAsnHisSerCysGluProCysGlnThrLeuAlaValArg :    445
         ||||||||||!!:|||!.!|||!!.|||! !|||! !||||||||||||||||||||
         lLeuGlyGlnAspArgHisAsnGlnSerTyrGluGlnCysGlnThrLeuAlaValArg
216972 : GCTCGGCCAGGACCGCCATAACCAGAGCTATGAGCAGTGCCAGACGCTGGCCGTGCGT : 216916

   446 : SerTyrArgLeuHisGluAlaPheSer-ProValSerTyrGlnHisAspLeu{A}  > :    463
         :!!|||! !|||||||||||||||||#  !:!!!!!|||||||||||||||{|}
         AlaTyrProLeuHisGluAlaPheSer#ValIleThrTyrGlnHisAspLeu{A}++
216915 : GCCTACCCCCTGCACGAGGCCTTCTCGCGTTATCACCTACCAGCACGACCTG{G}gt. : 216858

   464 : >>> Target Intron 11 >>>>  {la}LeuLeuArgLeuGlnGluAspAlaAsp :    472
                 95 bp           {||}|||:!!||||||||||||..!||||!!:
                              ++{la}LeuValArgLeuGlnGluSerAlaGlu
216857 : .........................ag{CT}CTGGTGCGCCTGCAGGAGAGCGCTGAA : 216739

   473 : GlySerCysAlaLeuLeuSerProTyrValGlnProValCysLeuProSerGlyAlaA :    492
         |||!!!|||||||! !! !||||||!:!||||||||||||||||||||||||.!!||||
         GlyCysCysAlaHisProSerProPheValGlnProValCysLeuProSerSerAlaA
216738 : GGCTGCTGCGCGCACCCGTCGCCTTTCGTTCAGCCAGTGTGCCTGCCGAGTAGCGCCG : 216679

   493 : laArgPro<-><->SerGluThrThrLeuCysGlnValAlaGlyTrpGlyHisGlnPh :    509
         ||! !|||      ||||||.!!.!!||||||:!!||||||||||||||||||||||!!
         laLeuProAlaGluSerGluAlaAlaLeuCysGluValAlaGlyTrpGlyHisGlnLe
216678 : CCCTCCCGGCCGAATCTGAAGCCGCGCTCTGCGAAGTGGCCGGCTGGGGTCACCAGTT : 216622

   510 : eGlu{G}  >>>> Target Intron 12 >>>>  {ly}AlaGluGluTyrAlaSe :    517
         .|||{|}           564 bp           {||} !!! ! !!|||:!!||
         uGlu{G}++                         ++{ly}ProGly***TyrSerSe
216621 : AGAA{G}gt.........................ag{GG}CCAGGGTAATATTCCAG : 216034

   518 : rPheLeuGlnGluAlaGlnValProPheLeuSerLeuGluArgCysSerAlaProAsp :    536
         |||||||||| !!.!!|||||||||.!!:!!  !! !||||||||||||||||||||
         rPheLeuGln***ThrGlnValProLeuIleHisProGluArgCysSerAlaProAsp
216033 : CTTCCTGCAGTAGACTCAGGTGCCGCTCATCCATCCGGAGCGCTGCTCCGCCCCCGAC : 215977

   537 : ValHisGlySerSerIleLeu-ProGlyMetLeuCysAlaGlyPheLeuGluGlyGly :    555
         |||||||||:!!:!!.!!:!!#|||||||||||||||||||||||||||||||||||
         ValHisGlyAlaAlaPheIle#ProGlyMetLeuCysAlaGlyPheLeuGluGlyGly
```

```
215976 : GTGCACGGAGCCGCCTTCATCCCCCGGCATGCTCTGCGCTGGCTTCCTCGAGGGTGGC : 215919

   556 : ThrAspAlaCysGln  >>>> Target Intron 13 >>>>  GlyAspSerGlyG :    565
         |||||||||||||||          91 bp          |||:!!||| !!|
         ThrAspAlaCysGln++                        ++GlyAsnSerArgG
215918 : ACTGACGCGTGCCAGgt.........................agGGTAACTCCAGGG : 215798

   566 : lyProLeuValCysGluAspGlnAlaAlaGluArgArgLeuThrLeuGlnGlyIleIl :    584
         ||||||||||||||||||||:!!.!!||||||||||!:!|||! !|||!:!||||||:!
         lyProLeuValCysGluAspGluThrAlaGluArgGlnLeuIleLeuArgGlyIleVa
215797 : GCCCTCTGGTGTGTGAGGATGAGACCGCAGAGCGCCAGCTCATCCTGCGAGGCATCGT : 215741

   585 : eSerTrpGlySerGlyCysGlyAspArgAsnLysProGlyValTyrThrAspValAla :    603
         !||||||||||||||||||||||||!.!:!!! !||||||||||||||||||||||||
         lSerTrpGlySerGlyCysGlyAspHisHisMetProGlyValTyrThrAspValAla
215740 : CAGCTGGGGTTCAGGTTGCGGCGACCACCACATGCCAGGTGTATACACCGACGTGGCC : 215684

   604 : TyrTyrLeuAlaTrpIleArgGluHisThrValSer :    615
          !!|||||||||||||||||||||||||||!.!|||
         AsnTyrLeuAlaTrpIleArgGluHisThrAlaSer
215683 : AACTACCTAGCCTGGATCCGGGAGCACACCGCTTCC : 215646
```

Figure S2: Highest scoring exonerate alignment of human factor 12 with the genome of *Tursiops truncatus*

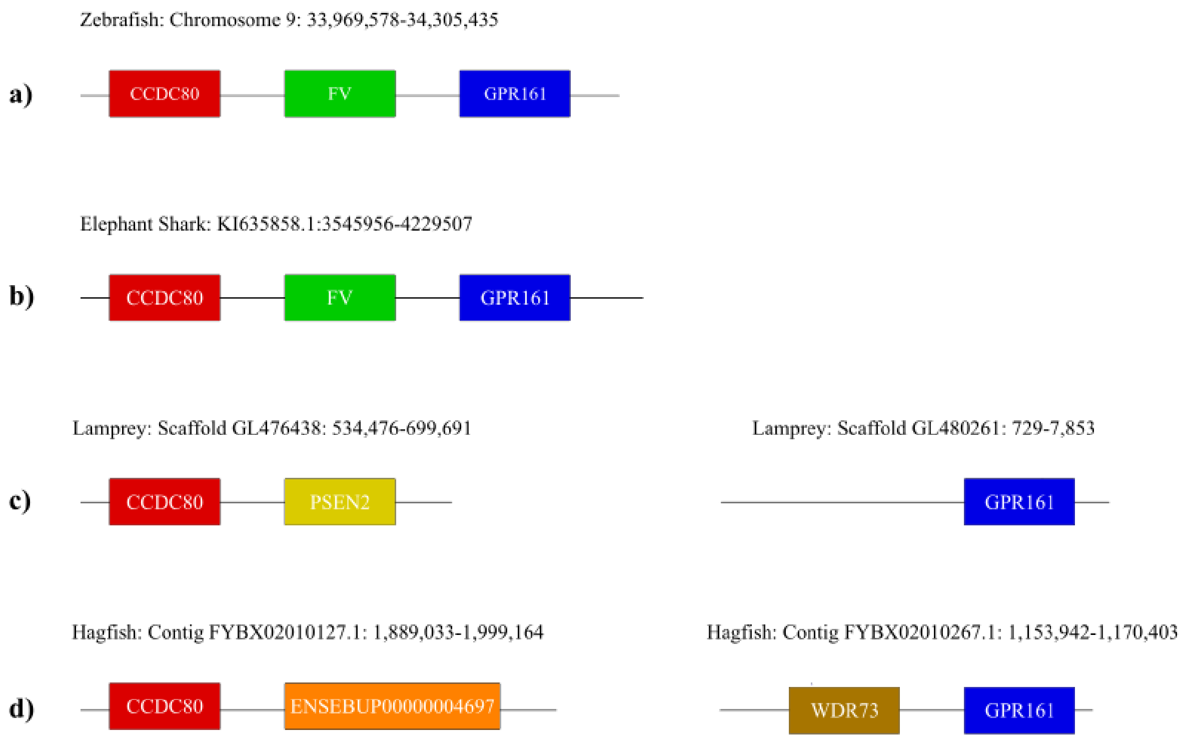

Figure S3: Synteny analysis results between a) D. rerio, b) C. milii, c) P. marinus and d) E. burgeri. The orthologues of CCDC80 and GPR161 are found in two different scaffolds/contigs in jawless vertebrates (c-d). In lamprey, GPR161 is the first gene in the scaffold GL480261.

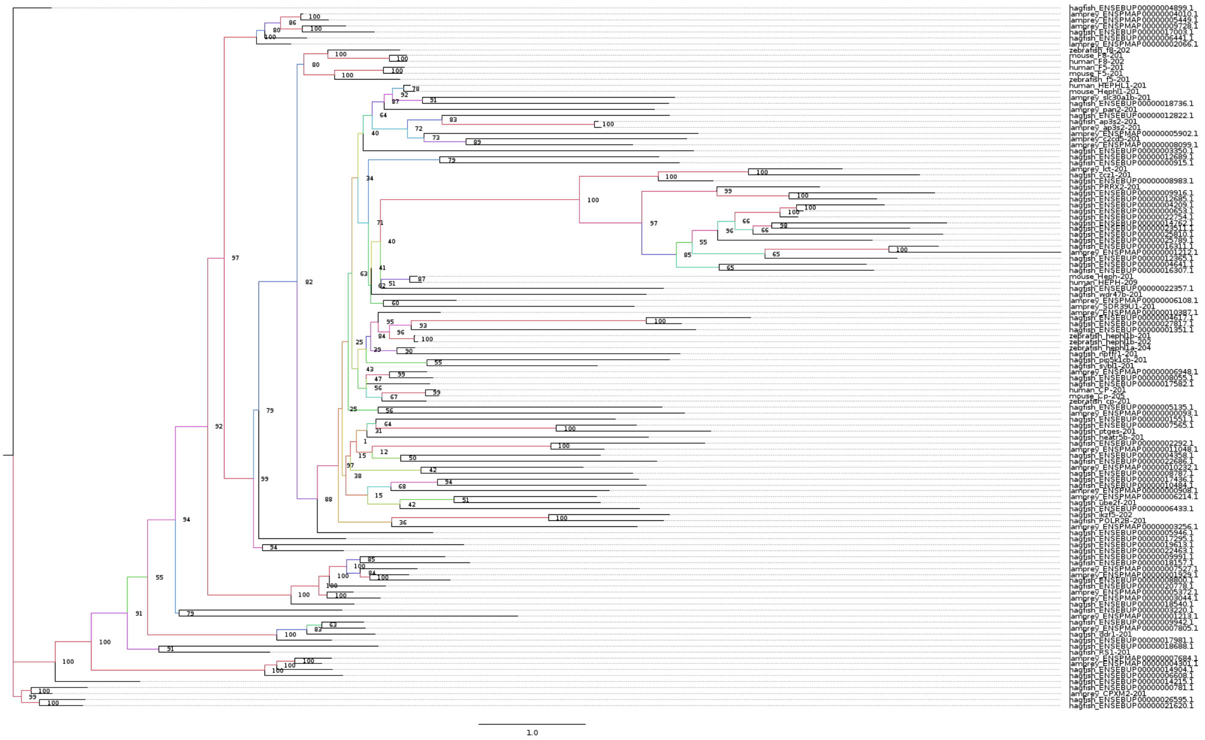

Figure S4: The complete phylogenetic tree of FV, FVIII, Hephaestin, Ceruloplasmin and their putative orthologues in jawless vertebrates.
